# The Significance of Proper Care for Textured Hair on Inpatient Psychiatric Units

**DOI:** 10.1159/sad/aesag006

**Published:** 2026-09-05

**Authors:** Devyn H. Zaminski, Archie Spindler, Derek Maas, Isabella Zappi, Sarah Commaroto, Efe Kakpovbia, Jerry Shapiro, Kristen I. Lo Sicco, Christin Drake

**Affiliations:** a NYU Grossman School of Medicine, New York, NY, USA; b University of Rochester School of Medicine, Rochester, NY, USA; c University of South Florida Morsani College of Medicine, Tampa, FL, USA; d The Ronald O. Perelman Department of Dermatology, NYU Grossman School of Medicine, New York, NY, USA; e Department of Dermatology, Bellevue Hospital, New York, NY, USA; f Department of Psychiatry, NYU Grossman School of Medicine, New York, NY, USA; g Women’s Health Services, Woodhull Hospital, Brooklyn, NY, USA

**Keywords:** Textured hair, Black hair, Curly hair, Equity of care, Dermatitis, Hair care, Hospital resources

## Abstract

**Background:**

Hair plays a pivotal role in identity, expression, and well-being. Patients with textured hair require specific products and practices to maintain scalp and hair health, including adapted washing frequency, moisturization, and hairstyle maintenance. However, inpatient psychiatric unit policies often restrict personal products due to safety and storage restrictions and may not otherwise supply appropriate alternatives.

**Summary:**

Without appropriate products, such as sulfate-free shampoos, leave-in conditioners, and moisturizing creams, inpatient psychiatric patients with textured hair are at risk of preventable complications, including seborrheic dermatitis, hair breakage, and traction alopecia. Because appearance and grooming are routinely assessed in psychiatric evaluations, inadequate hair care access may lead to misinterpretation of mental state, potentially delaying discharge and prolonging hospitalization. Additionally, inability to maintain one’s hair during hospitalization may cause emotional and psychological distress.

**Key Messages:**

Improper access to textured hair care products represents an inequity of inpatient psychiatric care that should be addressed. This article highlights the clinical, dermatologic, and psychosocial consequences of inadequate textured hair care in inpatient psychiatric settings and proposes a model to address this disparity through safe, inclusive hair care resources and staff education. Lived experience was not involved. Future research should quantify the impact of these initiatives.

## Introduction

Hair properties differ significantly across racial and ethnic groups, leading to varying requirements for maintenance and grooming [1, 2]. Many individuals of all racial and ethnic backgrounds have textured hair, which has intrinsic structural properties that cause it to be drier compared to non-textured hair, and thus requires less frequent washing and significantly more moisturizing [3, 4]. Proper hair care promotes scalp and hair health but also has a significant impact on identity, cultural practices, and overall well-being. This article is the product of a unique collaboration between psychiatrists and dermatologists to raise awareness regarding the importance of offering products for textured hair on inpatient psychiatric units.

## Background: Defining the Clinical Gap

Patients may lack the necessary products to care for textured hair during hospital psychiatric admissions. During the in-take process, for both voluntary and involuntary admissions, patients are required to relinquish all of their belongings to hospital staff. Toiletries are often prohibited for the duration of the patient stay, as they may contain alcohol or be packaged in containers that pose safety risks for individuals on the unit. While units allow personal items, volume restrictions for liquids can prevent access to hair care products that are often sold in large containers. These regulations are in place to ensure patient safety but have the unintended consequence of preventing patients with textured hair from maintaining scalp and hair health and basic grooming practices during their hospital stay. However, multiple studies have documented an overrepresentation of African Americans and Caribbean black patients, who more commonly have textured hair, in inpatient psychiatric settings compared to non-Hispanic white patients [5, 6]. As a result, the absence of providing textured hair products may be impacting a larger segment of the inpatient psychiatric population.

To conduct this review, a focused literature search was performed using PubMed and Google Scholar to identify studies relevant to textured hair care, scalp, and hair disorders associated with inadequate maintenance and the psychosocial and clinical implications of hair care in inpatient psychiatric settings. Search terms included combinations of “textured hair,” “black hair,” “African American hair,” “curly hair,” “traction alopecia,” “psychiatry,” “inpatient,” “grooming,” and “mental status examination.” Relevant references from selected articles were also reviewed to identify additional sources.

## Textured Hair and Care Needs

### Biology of Textured Hair

With diverse genetic qualities, hair ranges broadly in color, shape, and intrinsic physical properties [1]. These differences across populations have been identified, and immense strides have been taken to categorize hair types in order to guide proper care and maintenance [3]. The Andre Walker Hair Typing System categorized hair morphologies into types 1–4 designating straight, wavy, curly, and kinky hair, respectively, with additional subtypes for increasing shaft diameter [3]. Additionally, L’Oreal established a widely accepted comprehensive stratification of hair types consisting of eight distinct morphologies (I–VIII) based on four primary characteristics: curve diameter, curl index, number of kinks and twists, and number of waves [7].

Structural properties of hair are known to differ across ethnic populations [1]. For example, non-textured hair shafts are often cylindric, straight, and have an intermediate-sized diameter [4]. Conversely, textured hair is more commonly associated with classification types exhibiting curls, kinks, twists, and waves [1]. Hair shafts are typically elliptical or flattened and tightly spiraled with intermittent kinking [4, 8]. The coils and kinks can have variable shaft diameters, decreased tensile strength, and less moisture content which can cause increased breakage and potential for hair loss from general mechanical stress [3, 4].

### General Hair Care

Hair care regimens vary widely based on individual preferences and may be influenced by characteristics such as age, sex, gender, hair type, hairstyle, ethnicity, socioeconomic status, personal experiences, and evolving esthetic trends [3, 9]. General hair care regimens can include shampoos, conditioners, and/or fix-styling products (including longer term treatments such as relaxers, dyes, and bleaching chemicals) [9]. Shampoos are utilized to rinse dirt and oil from the scalp to promote a balanced microbiome while preventing local inflammation [10, 11]. Conditioners and other moisturizing products are important for replenishing moisture after shampooing or rehydrating dry or damaged hair [12]. These products can counteract frizz and friction brought on by anionic surfactants in shampoos to increase the softness, shine, and manageability of hair [12]. Routine cleansing with shampoos and conditioning thereafter are recommended for maintaining scalp and hair health [12, 13].

### Textured Hair Care Needs: Shampoo

Shampoos utilize multiple chemical compounds to cleanse the scalp and hair [11]. The primary active ingredients are surfactants which range in ionic composition (negatively charged anionic, positively charged cationic, amphoteric, and nonionic) and are utilized as detergents with varying strengths and properties according to hair needs [11]. Common additives can be incorporated for modification of shampoo properties. Keratolytics assist in removing excess surface scale and debris, thereby reducing dandruff, while antipruritic agents can reduce irritation or discomfort [11, 14]. For textured hair, it may be beneficial to avoid anionic surfactants, a harsher class of cleansing agent associated with more irritation, lipid removal, and dryness leading to an increased risk of hair breakage [15–17]. Common anionic surfactants, listed from harshest to mildest, include sodium lauryl sulfate, sodium laureth sulfate, sodium lauroyl sarcosinate, ammonium lauryl sulfate, sodium C14–16 olefin sulfonate, disodium laureth, and sulfosuccinate [18]. Gentler alternatives are decyl glucoside, cocamidopropyl betaine, and behentrimonium methosulfate [18]. The addition of ceramides in shampoo has been found to decrease breakage in textured hair [19].

The unique properties of textured hair as well as the influences of cultural traditions and popular trends have led to different practices among individuals with textured hair care [14, 20]. Research has found that African American women are more likely to wash their hair weekly or every 2 weeks, compared to Caucasian women who are more likely to wash their hair daily or every 2 days [21]. Variation in shampooing practices also exists within textured hair subsets. For example, textured hair individuals with looser curls, both men and women, may wash their hair multiple times in a given week, whereas those with tighter curls and more elaborate hairstyles may prefer less frequent cleansing, sometimes extending beyond once every 2 weeks [14]. Among individuals with textured hair in the UK, Dadzie and Salam [20] found a mean washing frequency of 17.9 days. More complex hair styling, such as braiding and weave application, was performed at an average frequency of 63 days with washing every 32.8 days [20]. These reports represent a difficult balance as decreased frequency of washing may cause scalp buildup, but increased frequency of washing can decrease scalp sebum and increase dryness potentially contributing to hair breakage [22].

### Textured Hair Care Needs: Conditioning

Following cleansing, conditioners are used to strengthen hairs with existing damage and to artificially replace sebum lost during washing, thereby improving hair fiber sheen, moisture, and ability to be styled [12, 14]. Conditioners are composed of various formulations of cationic surfactants, polymers, bodying agents, thickeners, and emollients [23]. Individuals with textured hair typically utilize conditioners containing proteins, silicone, and compounds to enhance hair softness and promote restoration of compromised fibers [14]. For convenience and additional moisture, conditioners or creams can be used on a regular basis on dry or damp hair – even in the absence of washing – and are recommended for drier hair types [14]. To preserve moisture and style integrity, individuals with textured hair may employ tiered routines following cleansing including a variety of additional moisturizing creams, oils, and pomades prior to styling [14].

### Textured Hair Care Needs: Styling

Styling practices for textured hair vary with individual preference, with some performing personal methods and others adopting frequent visits to salons for professional management [14]. Products such as gels, waxes, mousse, and sprays may be utilized to preserve a hairstyle for days to weeks; however, they may inadvertently cause dryness of hair shafts leading to breakage [14].

Additionally, individuals with textured hair are more likely to utilize chemical relaxers which break disulfide bonds in hair to straighten the curl pattern [14]. While often used to ease hair styling, chemical relaxers are associated with increased hair shaft fragility, scalp irritation or burns that result in scarring alopecia, acute kidney injury, and can contain endocrine-disrupting chemicals that can be hormonally active and carcinogenic [24]. Although applied every couple of months rather than employed in daily styling, this is important in understanding the various presentations and care requirements of textured hair.

Beyond chemical treatments, there exists a variety of physical techniques for hair styling. Existing coiling and kinking patterns can increase knotting and make textured hair challenging to maintain with standard combing techniques [4]. In addition to improving manageability with conditioning practices, the use of a wide-toothed comb or brush can reduce mechanical tugging that can break or uproot knotted textured hair. Complex hairstyles, such as braids, twists, or locs may be used to reduce time dedicated to daily styling and maintenance, increase length, or maintain density by retaining hair that has been shed from the scalp [14]. However, as previously discussed, patients with these hairstyles may delay frequency of scalp washing leading to irritation and risking the manifestation of other dermatologic consequences [20]. Furthermore, hairstyles traditionally utilized for textured hair, including cornrows, weaves, and locs, may exhibit increased tension on the hair follicle if styled too tightly [25]. Improper application, inadequate maintenance, or delayed removal of these hairstyles, particularly when combined with gels or waxes that help maintain high follicular tension, can lead to traction-induced alopecia [14, 25]. Understanding the care required to maintain these hairstyles is important as psychiatric patients may experience prolonged admissions and require the means to adequately manage or remove these hairstyles. Additional considerations for daily styling routines include variations in daily activity level, available time to dedicate to hair styling, and access to appropriate resources [22, 26].

Although traction alopecia is typically a chronic process rather than an acute condition, prolonged periods of sustained follicular tension during extended inpatient stays (weeks to months) may contribute to cumulative hair follicle damage, potentially resulting in clinically apparent alopecia during admission or manifesting later. Expert recommendations applicable to inpatient settings to reduce traction-related hair damage include loosening excessively tight styles, removing extensions and undoing styles at appropriate intervals, and alternating with lower tension hairstyles when possible [27]. However, evidence-based guidance regarding routine maintenance of these hairstyles, including optimal washing frequency and long-term care practices, remain limited. This gap highlights the need for further research to inform culturally appropriate, evidence-based recommendations for styles common among individuals with textured hair.

## Consequences of Improper Hair Care

### Dermatologic Considerations

Scalp health is important to the strength, quality, and growth of hair fibers [28]. Medically, inadequate cleansing and moisturization can lead to scalp buildup, irritation, and inflammation [22]. Additionally, this can create a high lipid environment from sebaceous gland production on the scalp, allowing *Malassezia* yeast to thrive, worsen inflammation, and cause dermatitis [29]. This results in a condition called seborrheic dermatitis, which clinically presents as itchy, scaly, erythematous plaques on the scalp that can roughen hair strands and decrease luster [28, 29]. Studies have found that individuals with textured hair have increased risk of developing seborrheic dermatitis [30]. Notably, dopamine-blocking antipsychotic medications commonly used in this population may further elevate risk by increasing sebum production [31]. Simple treatments exist for this condition; therefore, it is important for care teams to be able to identify it and act accordingly. Over-the-counter treatment options include shampoos with selenium sulfide or zinc pyrithione [29]. Severe cases may necessitate prescription treatments with active ingredients such as topical antifungals (ketoconazole 2% or ciclopirox 1% shampoos), calcineurin inhibitors, or corticosteroids [29]. Although these treatments are readily available through psychiatrist ordering or dermatology consultation, dermatologic conditions, including those of the scalp, remain underdiagnosed in individuals with serious mental illness and more proactive identification by inpatient care teams is needed [32].

When choosing hair care products, it is also important to consider the risk of allergic contact dermatitis, which presents as skin inflammation, itching, burning, and hair shedding [30]. Common irritants in hair care products include various surfactants (sodium laureth/lauryl sulfate, cocamidopropyl betaine, decyl/lauryl/coco/cetearyl glucosides), preservatives (methylisothiazolinone, formaldehyde), and fragrances [30]. Treatment should involve discontinuation of the offending product, topical anti-inflammatory treatment, and potential allergen patch testing to identify the causative agent [30].

### Clinical Implications

As previously discussed, the absence of proper hair care can result in increased hair breakage and a decline in appearance. In the inpatient psychiatric setting, this can have significant clinical consequences [12, 13]. On psychiatric wards, daily psychiatric assessments are routinely performed to determine an individual’s progress and inform clinical management, including duration of hospital stay. Daily assessments utilize mental status examinations to evaluate an individual’s appearance, grooming, and hygiene [33]. The rationale for including these components acknowledges that a patient’s ability to appropriately groom themselves and attend to their hygiene is determined by their level of behavioral and executive functioning. However, a lack of proper grooming resources can also result in a visible deterioration of appearance that is unrelated to psychiatric status. For patients with textured hair, the inability to access appropriate products could result in prolonged hospital stays.

Prior to admission to an inpatient psychiatric service, patients are often impacted by their psychiatric condition in such a way that they may have had a prolonged period of being unable to attend to their basic hygiene and grooming needs [34]. The hospitalization, therefore, may actually be a period during which restoration of hair and scalp health is required for a patient to return to an adequately groomed appearance.

Further, psychiatric disorders (i.e., anxiety disorders, obsessive-compulsive disorders, delusional disorders, substance use disorders) can have co-occurring dermatologic symptoms, most commonly pruritus [35]. This relationship can be explained by complex bidirectional interactions between the skin and central nervous system, the latter of which is often dysregulated and implicated in both psychiatric symptoms and itch perception [36]. If nonpsychiatric dermatologic abnormalities such as scalp dermatitis are not identified and addressed, this could also confound psychiatric assessments, potentially contribute to prolonged admissions, changes in care management, and inequities of care.

### Psychosocial Consequences

Hair styling holds a profound significance. In human society, it is a major form of expression and affects attraction and socialization [3, 37]. Hair is closely tied to confidence and identity, and its loss can cause significant emotional difficulty [38]. Among these difficulties associated with hair loss, psychosocial impacts are prominent and include lower self-esteem and self-image and an exacerbated sense of vulnerability [39]. Patients with various types of hair loss have consistently shown impairment on health-related quality of life measures, particularly in the emotional domain, with an increased risk for anxiety and depression [39–43].

Inadequate hair care can directly contribute to hair loss and can also cause psychosocial distress in the absence of an alopecia diagnosis [27]. Hair is rooted deeply in culture, and the inability to maintain it can make individuals feel stigmatized [44, 45]. This issue is intensified by structural inequalities associated with the discriminatory treatment of black individuals based on their hair [46].

## Solutions for Textured Hair Care in the Hospital Setting

The absence of resources or awareness regarding textured hair needs can have significant medical, emotional, and psychological consequences. In our clinical experience, there exists a paucity of guidance for textured hair care among inpatient psychiatric facilities. To combat this, we have started an initiative to increase education about textured hair care for psychiatry staff and patients and have advocated for inpatient psychiatric units to supply products designed for textured hair. We began this initiative within the NYC Health + Hospitals system at Bellevue and Woodhull, two of the largest safety-net public hospitals in the nation that provide care to underserved populations throughout New York City. A significant portion of the patient population in these hospitals has textured hair. Through our initiative, patients on inpatient psychiatric units have increasing access to products that are specifically designed for textured hair. These include a gentle shampoo free of harsh anionic substances and sulfates, a leave-in conditioner, a moisturizing cream, and a styling/twisting butter. We have also created an educational pamphlet with information about textured hair care needs, instructions for how to use each product, and a recommended hair regimen that can be catered to individual needs (Fig. 1). These pamphlets are used to educate hospital staff and are offered to patients on the units. Any patient, including those without textured hair, is able to access these products in an effort to promote inclusive hair care and patient autonomy. In order to accommodate safety regulations, each product is purchased and stored in gallon-sized containers. The products are distributed to patients in 1–3 oz volumes – depending on the product and the quantity/length of hair – by direct request or during daily community meetings.

**Fig. 1.**
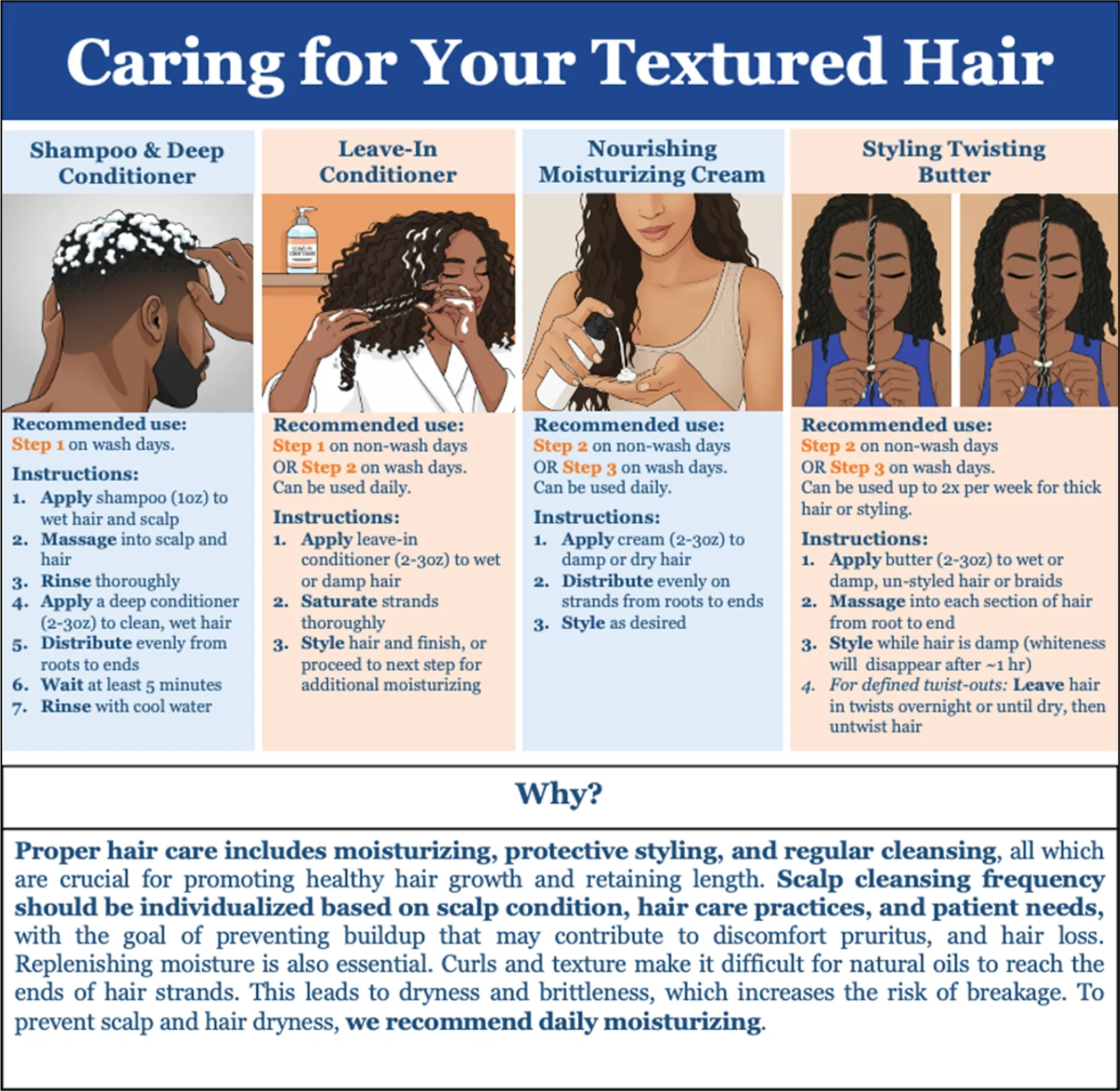
Educational pamphlet provided to staff and patients during distribution of textured hair products on psychiatric units. Contents include product instructions for use, two recommended hair care regimens for textured hair, and teaching points describing the importance of proper hair care.

In the few months since implementing our program, patients and staffs have expressed overwhelmingly positive feedback and no cases of adverse events. Staffs have shared improvements in patient affect and disposition after using the products and note that patients have been displaying greater personal pride. Patients have also made an effort to express their gratitude to our staff for the educational pamphlet and have been dedicating more time to self-care.

## Conclusions and Perspectives

Hair is an important part of an individual’s identity and self-esteem, and loss of hair or increased scalp symptoms may lead to distress, particularly during vulnerable periods such as inpatient psychiatric admissions [39]. Recognizing that different patient populations have different hair needs is vital for equity of care. We hope to spread awareness about this unmet need within our healthcare system, encourage advocacy for supplying textured hair care resources on psychiatric units, and offer an example of a solution that has worked in our hospitals. We appreciate that further research and logistical coordination may be necessary for expansion of this initiative to other hospitals. Our team plans to contribute to this space by performing survey studies of patients and staff to quantify the impact of this initiative beyond subjective measures. Ultimately, future studies should include patient-reported outcomes assessing satisfaction and perception of self, staff-observed outcomes of any changes in grooming and appearance assessment scores, medical outcomes such as rates of seborrheic dermatitis, allergic contact dermatitis, and patient-reported scalp discomfort, and comparison of hospital admission duration.

## Conflict of Interest Statement

Kristen I. Lo Sicco is an Editor of *Skin Appendage Disorders*, an investigator for Pfizer, a consultant for Pfizer, Lilly, Ro, Veradermics, and Acquis, a recipient of Dermatology Grand Rounds speaker honoraria, and a board member of the Scarring Alopecia Foundation and American Hair Research Society. Kristen I. Lo Sicco was a member of the journal’s Editorial Board at the time of submission. All other authors declare no potential conflicts of interest with respect to the research, authorship, and/or publication of this article.

## Funding Sources

This study received no funding.

## Author Contributions

Christin Drake, Kristen I. Lo Sicco, and Efe Kakpovbia contributed to this manuscript’s idea conception and supervised all aspects of manuscript writing, editing, and revisions. Devyn H. Zaminski and Archie Spindler led the literature search, original manuscript writing and revising, and figure creation. Derek Maas and Isabella Zappi assisted with original manuscript writing. Jerry Shapiro contributed to manuscript editing and revisions. Sarah Commaroto created the graphical abstract and contributed to manuscript editing and revisions. All authors contributed significantly to the production of this manuscript.
